# Reactivity of EEG patterns is a crucial indicator to determine the EEG is not ictal: A case of topiramate overdose

**DOI:** 10.1002/epd2.20298

**Published:** 2024-10-05

**Authors:** Philippe Gélisse, Arielle Crespel

**Affiliations:** ^1^ Epilepsy Unit Gui de Chauliac Hospital Montpellier France; ^2^ Research Unit (URCMA: Unité de Recherche sur les Comportements et Mouvements Anormaux) INSERM, U661 Montpellier France

Hleuhel et al. reported the case of baclofen intoxication with generalized periodic discharges at 2.6 Hz, fulfilling the Salzburg criteria for definite nonconvulsive status epilepticus (NCSE).^1^ However, auditory stimulation led transiently to the termination of the epileptiform activity and the authors concluded that the EEG pattern was the consequence of a toxic encephalopathy and that the strict application of the Salzburg criteria for NCSE may lead to an overestimation of NCSE in baclofen intoxication.

Overdose of topiramate may result in a coma, seizures, hemodynamic instability, and severe metabolic acidosis.^2^ We report a 10.5‐year‐old boy who attempted suicide with 700 mg of topiramate (his mother's antiseizure medication) resulting in a confusional state and low‐serum bicarbonate on a blood test (16 mmol/L). His EEG showed when his eyes were closed, a bilateral rhythmic activity at 4.5 Hz with a sinusoidal aspect (Figure 1A; Supporting Information). The anterior rhythmic theta waves disappeared upon eye‐opening but were still present on the posterior regions, especially on the left side (Figure 1B; Supporting Information). The reactivity to eye‐opening eliminates an absence status epilepticus (ASE). The patient recovered spontaneously and the control EEG performed 3 days later was normal.

**FIGURE 1:**
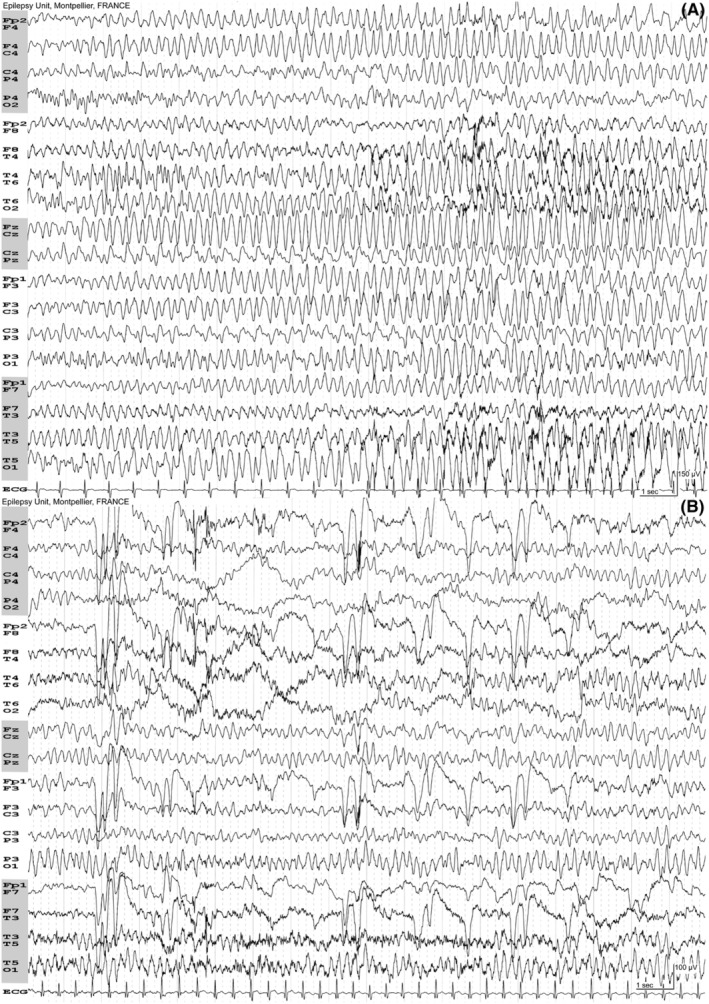
(A) Recording at 15 mm/s, 150 μV/cm. Patient's eyes are closed. Bilateral anterior rhythmic theta waves at 4.5 Hz. Note the sinusoidal aspect. (B) Recording at 15 mm/s; 100 μV/cm. Patient's eyes are open with eyelid closing artifacts on the frontopolar leads. Note the reactivity. The anterior theta waves disappear upon eye‐opening but are still present on the posterior regions with predominance on the left hemisphere.

EEG reactivity refers to a change in the EEG background activity in response to stimulation (change in amplitude and/or frequency, including attenuation of activity),^3^ and is considered a marker of good prognosis in comatose patients, especially after a cardiac arrest.^3^ With the strict application of the Salzburg criteria for NCSE,^4^ the EEG of our patient with continuous bilateral epileptiform discharges >2.5 Hz in association with a confusional state corresponded to NCSE. There was also a fluctuation of the morphology of the pattern with a sinusoidal aspect, but unusual for an epileptic activity. This EEG pattern with a sinusoidal aspect differentiates it from an epileptic seizure, but more importantly, the reactivity to eye‐opening allowed us definitively to retain a toxic encephalopathy and no antiseizure medication has been given. Due to self‐perpetuating processes and the failure of self‐terminating mechanisms, NCSE epilepticus is unlikely to cease transitory when patients open their eyes, except in patients with epilepsy with eyelid myoclonia (Jeavons syndrome) where the discharges in the case of ASE may be fragmented by eyes opening.^5^ Testing of the reactivity is an easy and safe test that must always be carried out when there is any doubt regarding an NCSE.^6^

We confirm that we have read the Journal's position on issues involved in ethical publication and affirm that this report is consistent with those guidelines.

## AUTHOR CONTRIBUTIONS

Philippe Gélisse: Conceptualization, Writing—original draft, Writing—review & editing. Arielle Crespel: Writing—review & editing. All co‐authors have been substantially involved in the study and preparation of the manuscript. No undisclosed persons have had a primary role in the study or manuscript preparation.

## CONFLICT OF INTEREST STATEMENT

Dr. Gélisse received support for teaching programs from UCB, Eisai and royalties for publishing from John Libbey Eurotext. Dr. Crespel received support for teaching programs from UCB, Eisai and royalties for publishing from John Libbey Eurotext. She was an advisory board member for Eisai‐France.

## Supporting information


Data S1.

Data S2.
