# Supplementary material for: Reactivity of EEG patterns is a crucial indicator to determine the EEG is not ictal: A case of topiramate overdose
Source: Epileptic Disord. 2024 Oct 5;27(1):137–8. doi: 10.1002/epd2.20298 (PMC11829620; doi:10.1002/epd2.20298)
Supplement: Supplementary file 1 — Data S1. [file EPD2-27-137-s001.docx]

**Test yourself**

**Answers:**

1. B, C, D

2. A, C
