# Supplementary material for: Reactivity of EEG patterns is a crucial indicator to determine the EEG is not ictal: A case of topiramate overdose
Source: Epileptic Disord. 2024 Oct 5;27(1):137–8. doi: 10.1002/epd2.20298 (PMC11829620; doi:10.1002/epd2.20298)
Supplement: Supplementary file 2 — Data S2. [file EPD2-27-137-s002.pptx]

## Slide 1
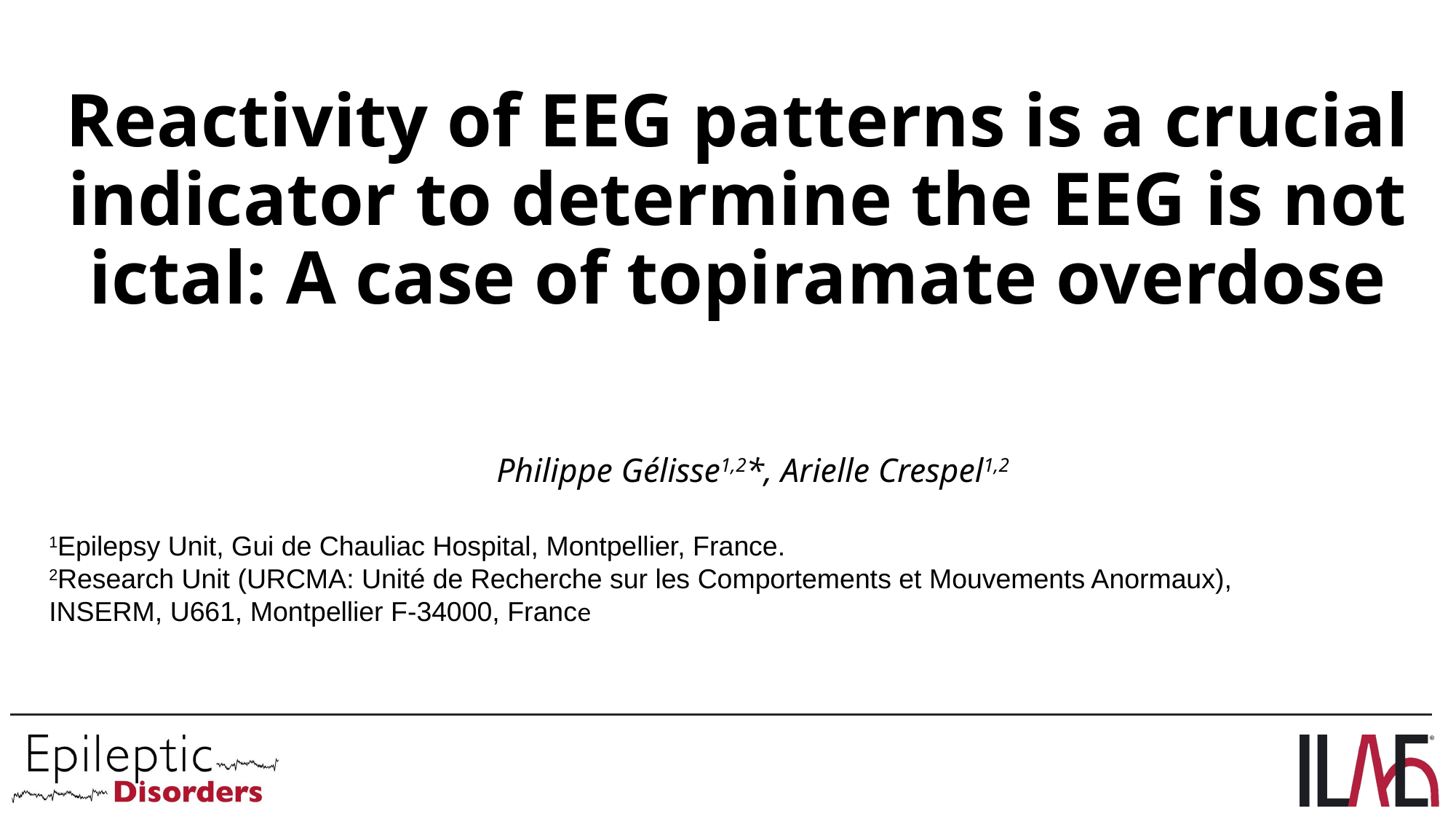

# Reactivity of EEG patterns is a crucial indicator to determine the EEG is not ictal: A case of topiramate overdose
Philippe Gélisse1,2*, Arielle Crespel1,2
1Epilepsy Unit, Gui de Chauliac Hospital, Montpellier, France.
2Research Unit (URCMA: Unité de Recherche sur les Comportements et Mouvements Anormaux), INSERM, U661, Montpellier F-34000, France

## Slide 2
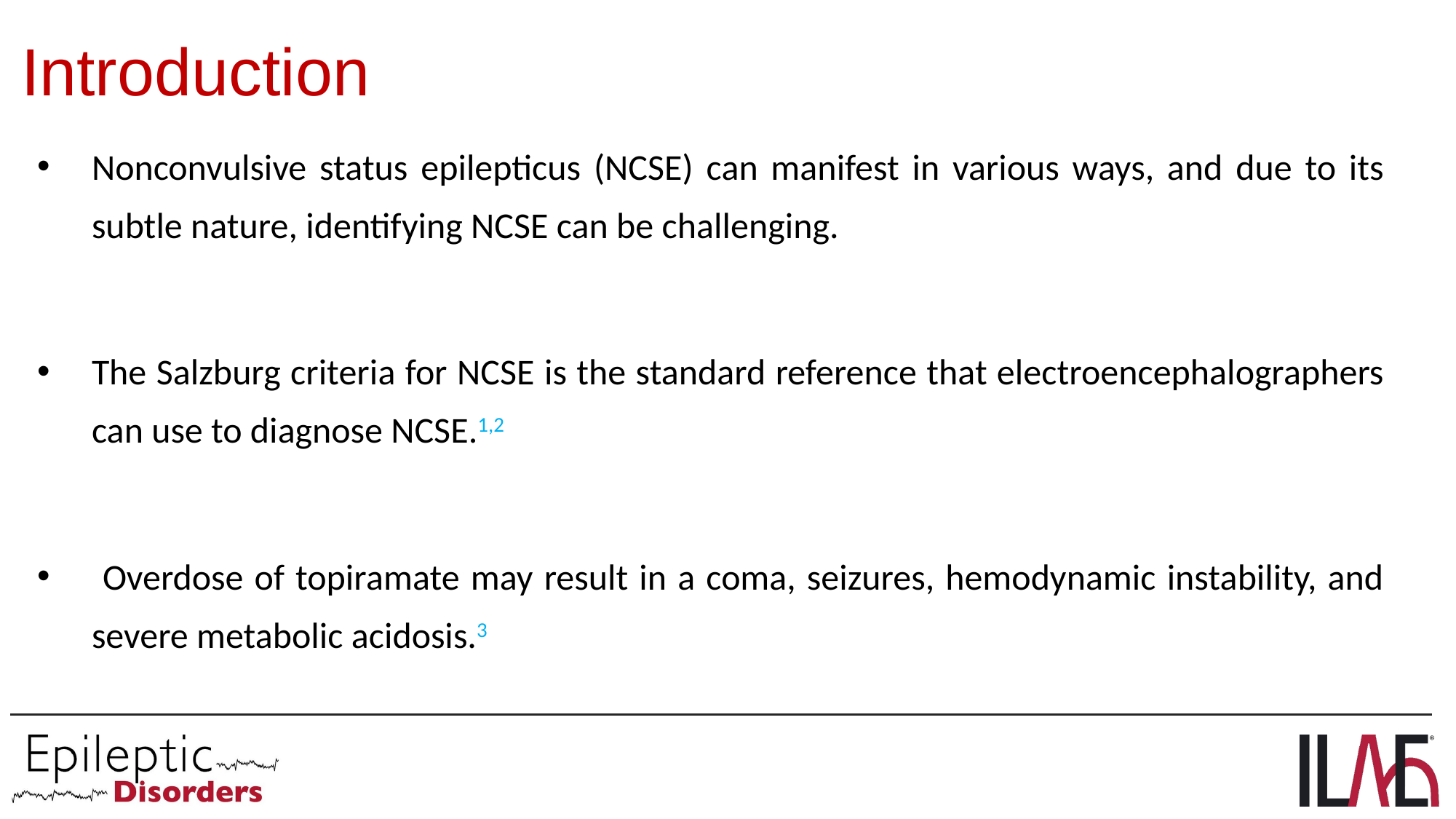

Introduction
Nonconvulsive status epilepticus (NCSE) can manifest in various ways, and due to its subtle nature, identifying NCSE can be challenging.
The Salzburg criteria for NCSE is the standard reference that electroencephalographers can use to diagnose NCSE.1,2
 Overdose of topiramate may result in a coma, seizures, hemodynamic instability, and severe metabolic acidosis.3

## Slide 3
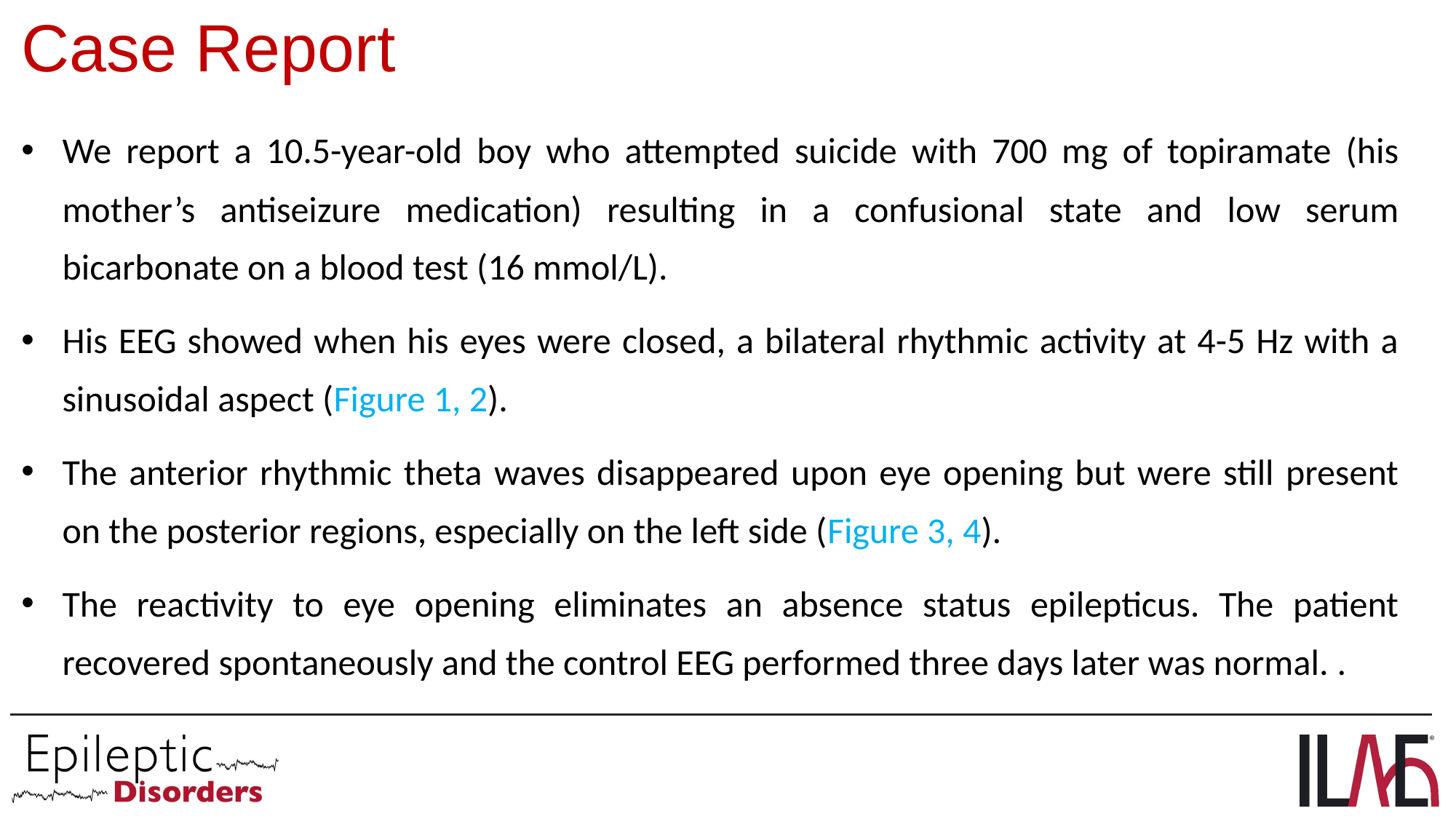

Case Report
We report a 10.5-year-old boy who attempted suicide with 700 mg of topiramate (his mother’s antiseizure medication) resulting in a confusional state and low serum bicarbonate on a blood test (16 mmol/L).
His EEG showed when his eyes were closed, a bilateral rhythmic activity at 4-5 Hz with a sinusoidal aspect (Figure 1, 2).
The anterior rhythmic theta waves disappeared upon eye opening but were still present on the posterior regions, especially on the left side (Figure 3, 4).
The reactivity to eye opening eliminates an absence status epilepticus. The patient recovered spontaneously and the control EEG performed three days later was normal. .

## Slide 4
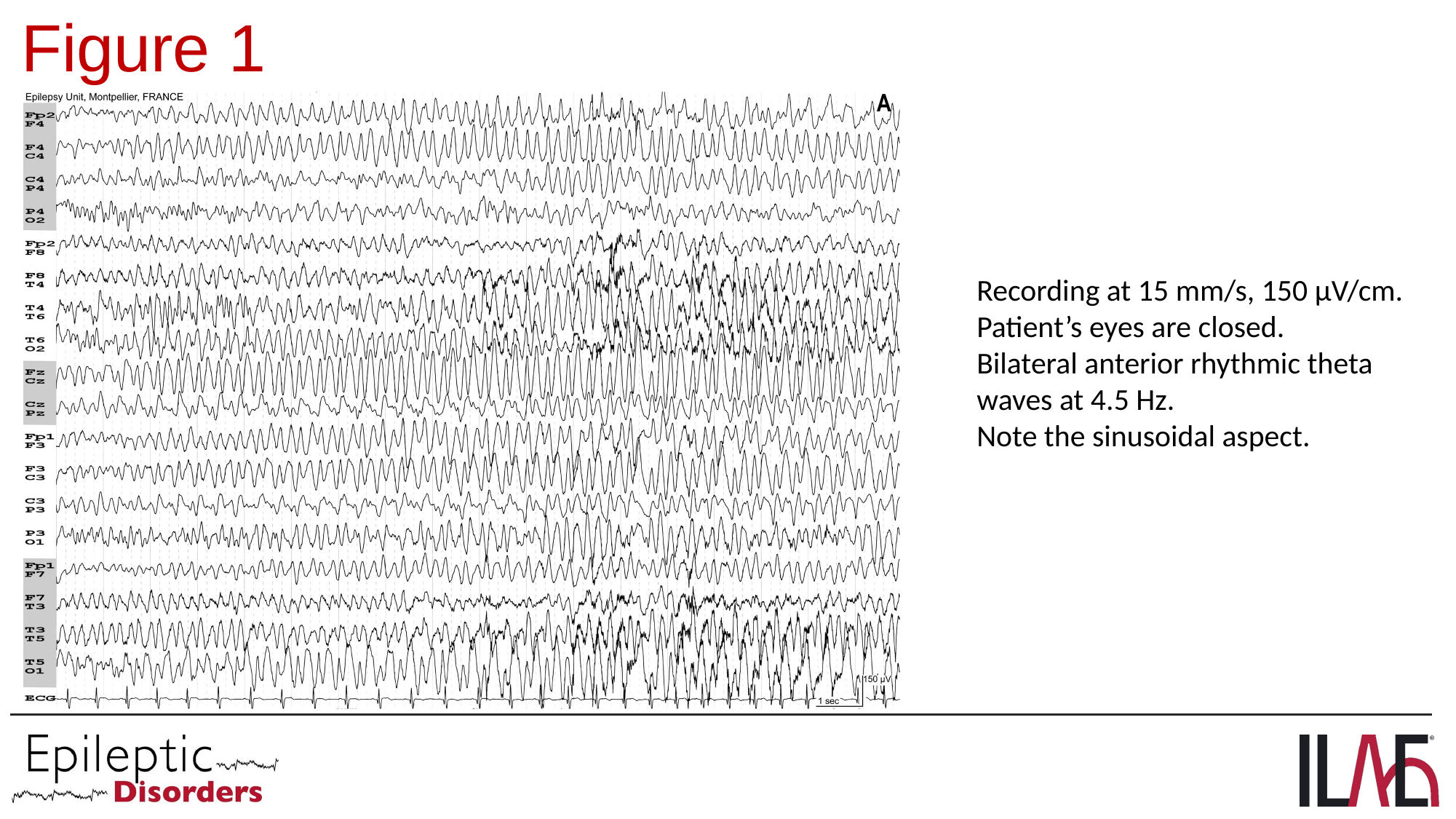

Figure 1
Recording at 15 mm/s, 150 µV/cm.
Patient’s eyes are closed.
Bilateral anterior rhythmic theta waves at 4.5 Hz.
Note the sinusoidal aspect.

## Slide 5
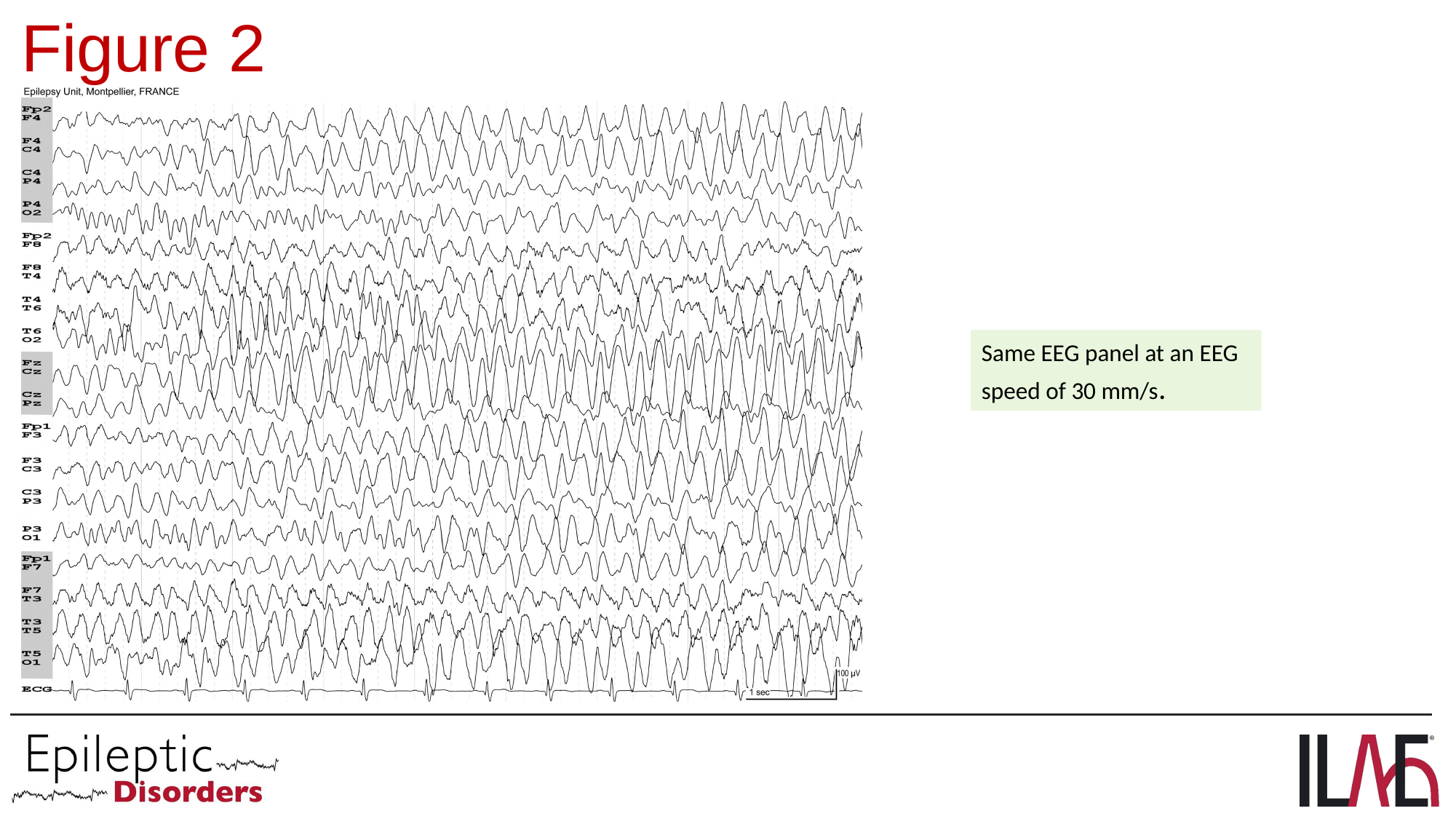

Figure 2
Same EEG panel at an EEG speed of 30 mm/s.

## Slide 6
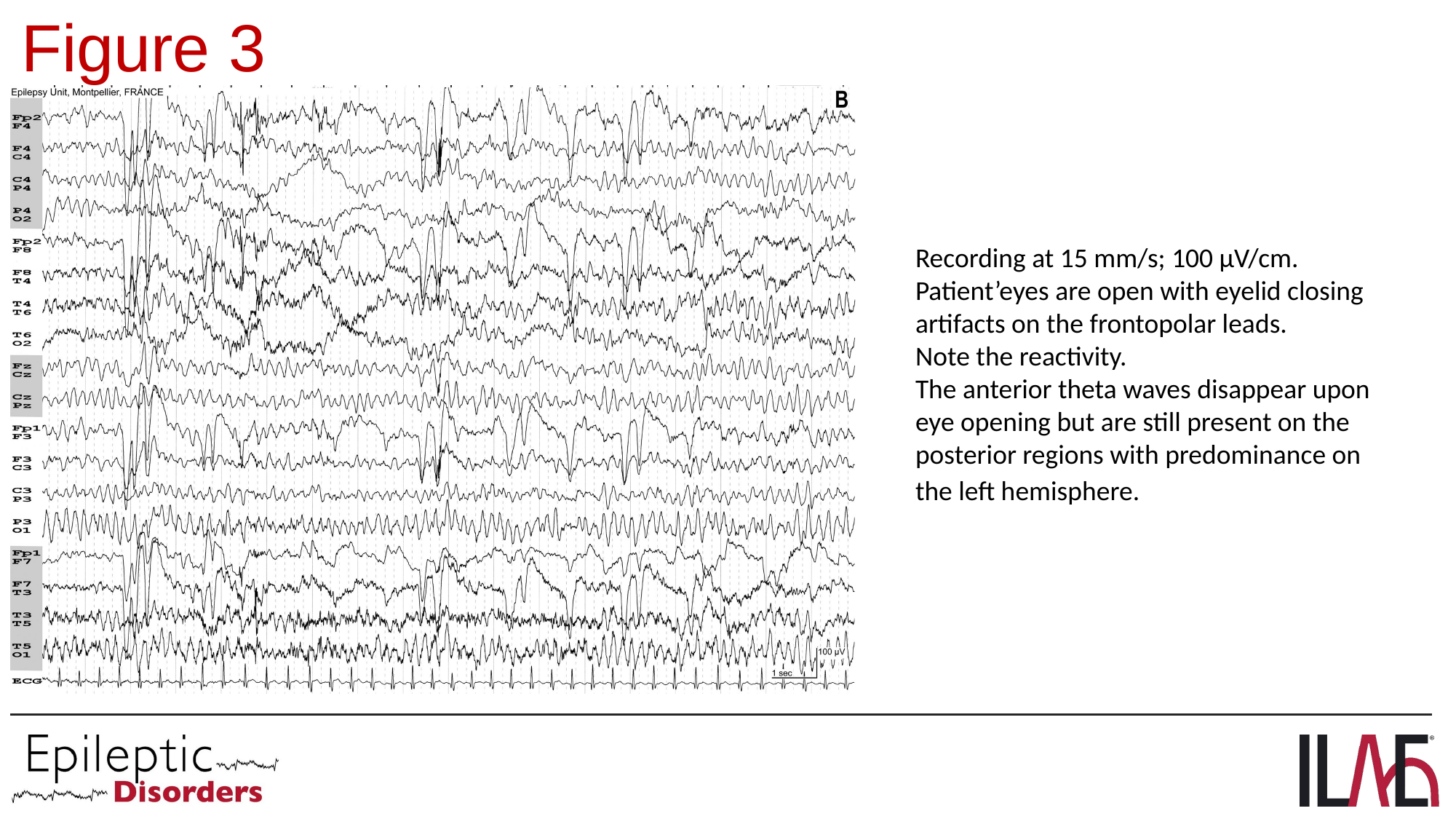

Figure 3
Recording at 15 mm/s; 100 µV/cm. Patient’eyes are open with eyelid closing artifacts on the frontopolar leads.
Note the reactivity.
The anterior theta waves disappear upon eye opening but are still present on the posterior regions with predominance on the left hemisphere.

## Slide 7
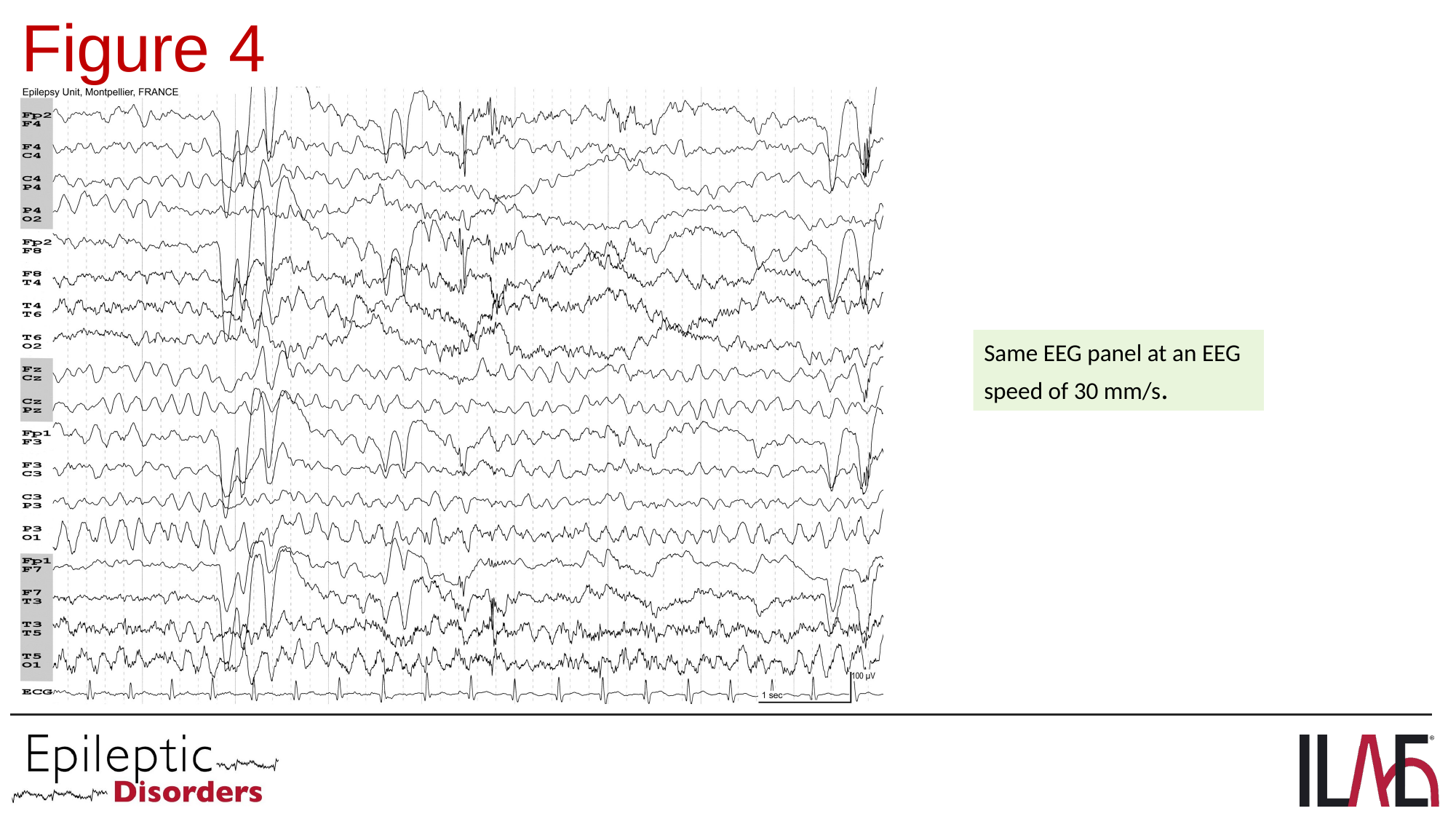

Figure 4
Same EEG panel at an EEG speed of 30 mm/s.

## Slide 8
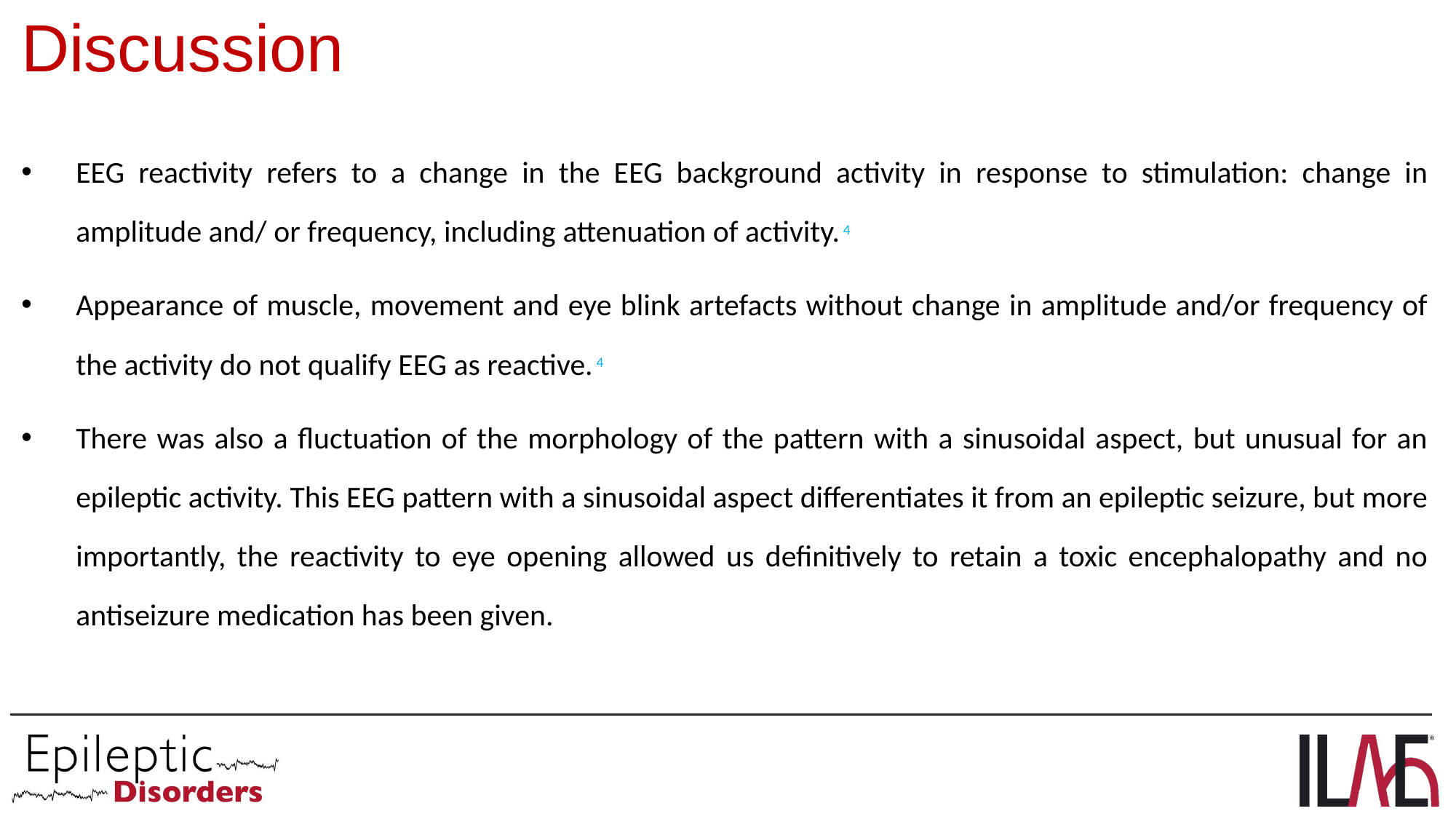

Discussion
EEG reactivity refers to a change in the EEG background activity in response to stimulation: change in amplitude and/ or frequency, including attenuation of activity. 4
Appearance of muscle, movement and eye blink artefacts without change in amplitude and/or frequency of the activity do not qualify EEG as reactive. 4
There was also a fluctuation of the morphology of the pattern with a sinusoidal aspect, but unusual for an epileptic activity. This EEG pattern with a sinusoidal aspect differentiates it from an epileptic seizure, but more importantly, the reactivity to eye opening allowed us definitively to retain a toxic encephalopathy and no antiseizure medication has been given.

## Slide 9
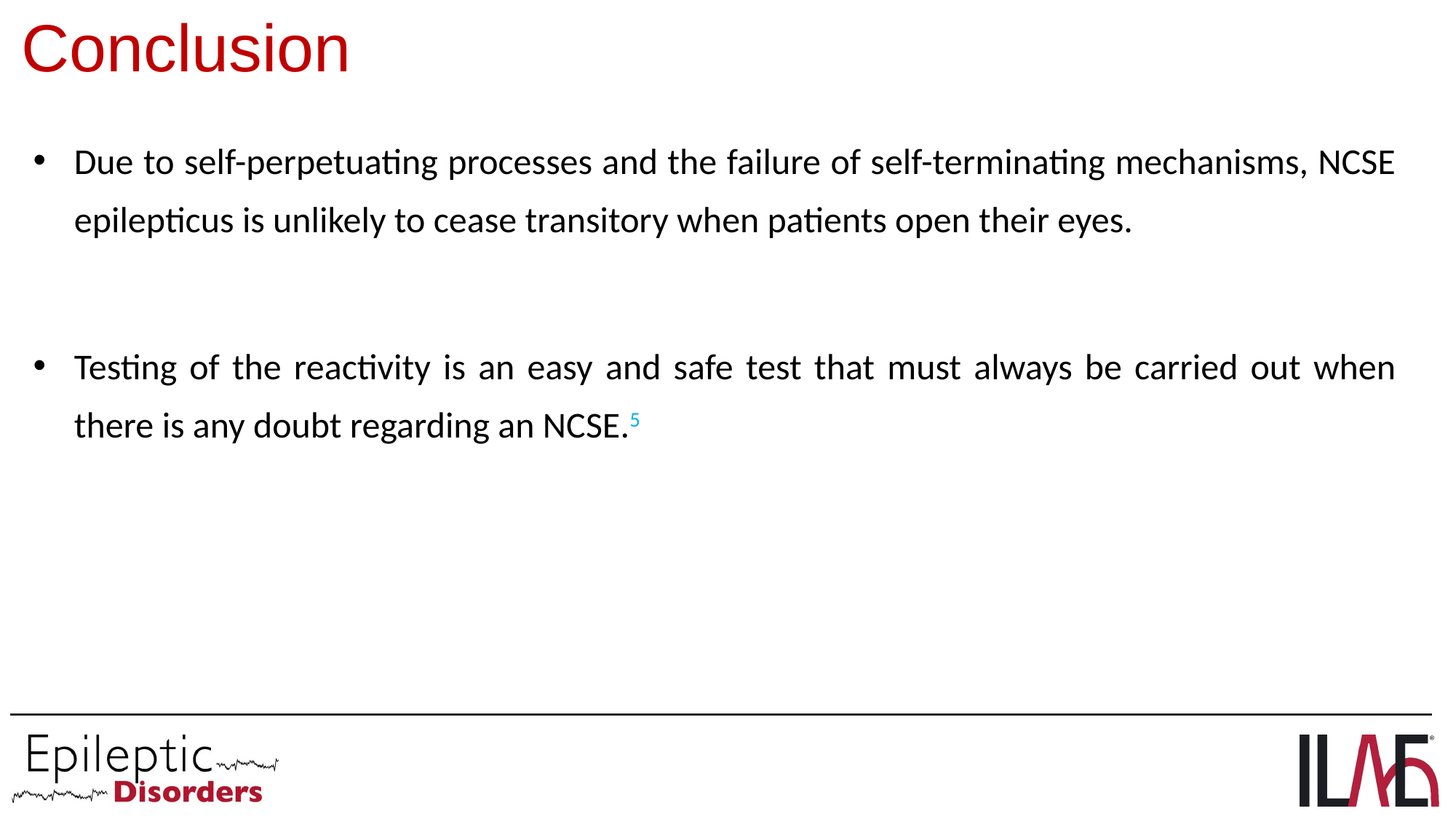

Conclusion
Due to self-perpetuating processes and the failure of self-terminating mechanisms, NCSE epilepticus is unlikely to cease transitory when patients open their eyes.
Testing of the reactivity is an easy and safe test that must always be carried out when there is any doubt regarding an NCSE.5

## Slide 10
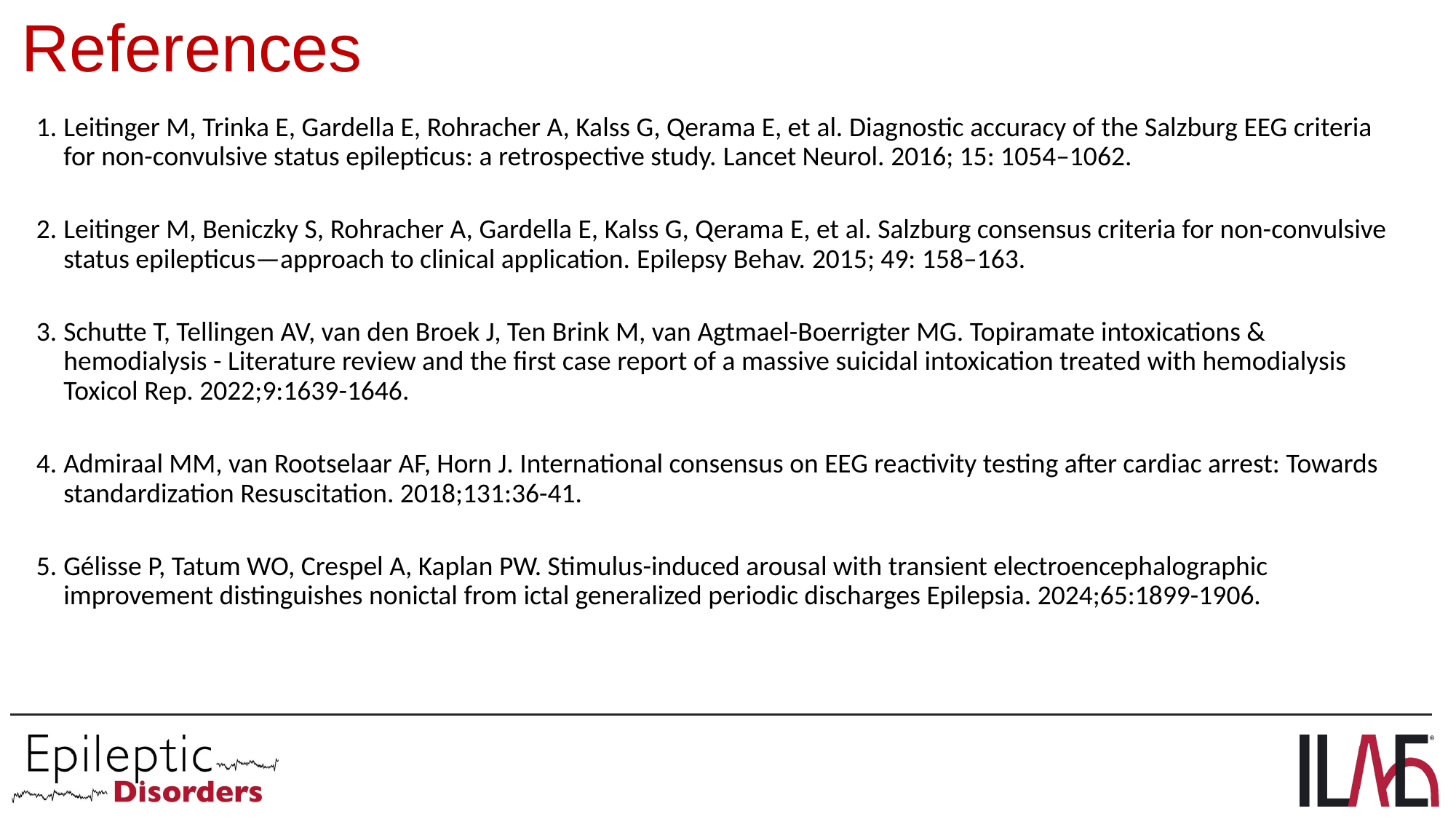

References
Leitinger M, Trinka E, Gardella E, Rohracher A, Kalss G, Qerama E, et al. Diagnostic accuracy of the Salzburg EEG criteria for non-convulsive status epilepticus: a retrospective study. Lancet Neurol. 2016; 15: 1054–1062.
Leitinger M, Beniczky S, Rohracher A, Gardella E, Kalss G, Qerama E, et al. Salzburg consensus criteria for non-convulsive status epilepticus—approach to clinical application. Epilepsy Behav. 2015; 49: 158–163.
Schutte T, Tellingen AV, van den Broek J, Ten Brink M, van Agtmael-Boerrigter MG. Topiramate intoxications & hemodialysis - Literature review and the first case report of a massive suicidal intoxication treated with hemodialysis Toxicol Rep. 2022;9:1639-1646.
Admiraal MM, van Rootselaar AF, Horn J. International consensus on EEG reactivity testing after cardiac arrest: Towards standardization Resuscitation. 2018;131:36-41.
Gélisse P, Tatum WO, Crespel A, Kaplan PW. Stimulus-induced arousal with transient electroencephalographic improvement distinguishes nonictal from ictal generalized periodic discharges Epilepsia. 2024;65:1899-1906.
